# The corpus callosum in people with congenital adrenal hyperplasia (CAH)

**DOI:** 10.1038/s41598-025-88870-z

**Published:** 2025-02-04

**Authors:** Eileen Luders, Debra Spencer, Caitlin Dale, Ieuan A. Hughes, Ajay Thankamony, Umasuthan Srirangalingam, Helena Gleeson, Helen Simpson, Melissa Hines, Florian Kurth

**Affiliations:** 1 Department of Women’s and Children’s Health, Uppsala University, Uppsala, Sweden; 2 School of Psychology, University of Auckland, Auckland, New Zealand; 3 Laboratory of Neuro Imaging, USC Stevens Institute for Neuroimaging and Informatics, Keck School of Medicine of USC, University of Southern California, Los Angeles, CA USA; 4 Department of Psychology, University of Cambridge, Cambridge, UK; 5 Department of Paediatrics, Addenbrooke’s Hospital, University of Cambridge, Cambridge, UK; 6 The Weston Centre for Paediatric Endocrinology and Diabetes, Addenbrooke’s Hospital, University of Cambridge, Cambridge, UK; 7 Department of Endocrinology and Diabetes, University College Hospital London, London, NW1 2BU UK; 8 Queen Elizabeth Hospital, Birmingham, UK; 9 Department of Diagnostic and Interventional Radiology, Jena University Hospital, Jena, Germany

**Keywords:** Androgens, Corpus callosum, Corticosteroid, Development, Magnetic resonance imaging, Sex, Neuroscience, Diseases of the nervous system

## Abstract

Congenital Adrenal Hyperplasia (CAH) is a group of genetic disorders that affect the adrenal glands. CAH manifests in abnormal levels of cortisol and androgens and is accompanied by white matter alterations. However, no CAH study has specifically targeted the corpus callosum, the brain’s largest white matter fiber tract. To bridge that gap in the literature, we investigated callosal morphology in 53 individuals with CAH and 53 matched controls (66 women, 40 men). In addition to calculating areas for seven callosal subsections, we estimated the callosal thickness at 100 equidistant points. All statistical analyses were conducted while co-varying for age and total brain volume and applying corrections for multiple comparisons. There were no significant effects of biological sex and no significant group-by-sex interactions. However, there was a significant effect of group, both for area measures and thickness estimates, indicating smaller dimensions within the callosal splenium and isthmus in people with CAH. Our findings corroborate previous studies highlighting white matter alterations in CAH and may suggest that callosal integrity is compromised due to potentially adverse effects of glucocorticoids, a standard treatment for both men and women with CAH.

## Introduction

Congenital adrenal hyperplasia (CAH) encompasses a group of genetic disorders that affect the adrenal glands, leading to abnormal levels of cortisol and androgens^1,2^. More specifically, the primary cause of CAH is a mutation in the gene CYP21A2, which encodes the enzyme 21-hydroxylase. Among other things, this enzyme is critical for the production of cortisol in the adrenal glands. Given that the enzyme is deficient in CAH, the synthesis of cortisol is impaired^3^. As a result, cortisol precursors are diverted into the production of androgens. Altogether, this results in decreased levels of prenatal cortisol in males and females as well as in increased levels of prenatal androgens in females only (the excess of adrenal androgens down-regulates the production of testicular androgens in males, so they do not have a net surplus of prenatal androgens)^4–8^.

After birth, both girls and boys with CAH are treated by administering glucocorticoids, which increases cortisol levels in males and females as well as decreases androgen levels in females. However, the treatment can result in excessively high glucocorticoid levels, potentially altering brain development by affecting neuronal maturation and myelination^9^. This may lead to changes in the micro- and macrostructure of the brain, particularly affecting the white matter, which is made up of myelinated nerve fibers. Indeed, prior studies have repeatedly reported white matter alterations, such as white matter hyperintensities, lesions, reduced volumes or compromised integrity, in individuals with CAH^8–19^.

The corpus callosum is the largest white matter fiber structure in the human brain but, to our knowledge, no CAH study has specifically focused on the corpus callosum. Notwithstanding, there are CAH studies that detected abnormalities within the corpus callosum (among other regions)^10,13,14,17,20,21^, including callosal agenesis^18,22^. However, some of this research was based on single cases and/or conducted in individuals who suffered from several co-morbidities. Moreover, a few studies did not include male participants; others lacked a control group. In general, sample sizes were small, perhaps compromising statistical power. Thus, to expand an understudied field of research, we set out to explicitly focus on the corpus callosum while trying to mitigate a number of possible pitfalls: We compiled a relatively large sample of individuals with CAH and well-matched controls (*n* = 106). We included both women and men (66 females / 40 males), and we excluded participants with neurological or psychiatric disorders.

As summarized elsewhere^23^, the corpus callosum is topographically organized. This means that fibers connecting posterior regions of the brain travel primarily through caudal callosal sections, while those connecting anterior regions travel primarily through rostral callosal sections. Therefore, it seems appropriate to obtain region-specific callosal measures, rather than investigating the corpus callosum as a whole. In order to create macroscopic subregions of the corpus callosum, various parcellations schemes have been developed. The Witelson scheme has been widely used in the neuroscience community, either to analyze callosal morphology and/or as a frame of reference when describing study findings^24^. Consequently, in the present study, we also applied the Witelson scheme which divides the corpus callosum into seven vertical partitions based on defined fractions of its maximum anterior-posterior length. In addition, to increase the regional specificity further, we applied a well-validated computational method capturing callosal distances (inferior to superior) at 100 equidistant points across the callosal surface. This resulted in 100 callosal thickness estimates at sub-voxel resolution. The method has been successfully applied in a wide range of studies, including studies on biological sex^25,26^ and developmental stages^27,28^, as well as various clinical conditions^29–43^.

## Methods

### Study sample

The sample consisted of 53 individuals (33 women and 20 men) with classic 21-Hydroxylase-Deficient CAH^1,2^ and 53 controls (33 women and 20 men), ranging between 18 and 46 years of age. Of the 53 individuals with CAH, 29 presented with a salt-wasting phenotype and 18 with a simple virilizing phenotype; the remaining 6 individuals with CAH did not have information on the phenotype. Individuals with CAH were matched pair-wise to controls with respect to sex, age, education, and verbal intelligence, as determined using the Advanced Vocabulary Test^44^. Table 1 provides descriptive statistics on age, education, and verbal intelligence for each subgroup. All participants were required to be free from neurological or psychiatric disorders and to have no contraindications to neuroimaging. Approval for the study was obtained from an NHS Research Ethics Committee and the Health Research Authority in the United Kingdom (15/EM/0532) as well as the Ethics Committee at the University of Auckland in New Zealand (020825). All participants provided their informed consent, and all experiments were performed in accordance with relevant guidelines and regulations.

**Table 1:** Group-specific descriptive statistics.

	Women with CAH	Control Women	Men with CAH	Control Men
N	33	33	20	20
Salt-wasting*	20	–	9	–
Simple virilizing*	10	–	8	–
Age (in years)	31.1 ± 8.6 [18.3–45.7]	31.8 ± 8.5 [18.3–45.3]	28.5 ± 6.6 [19.3–43.4]	27.9 ± 5.5 [19.4–40.8]
Verbal Intelligence	6.3 ± 2.6 [1.5–11.2]	6.3 ± 2.3 [1.8–11.0]	5.6 ± 3.4 [2.0–12.5]	6.4 ± 3.1 [-1.0–13.5]
Education**	4.0 ± 1.3	4.1 ± 1.3	3.8 ± 1.4	3.9 ± 1.2

* Phenotype information (salt-wasting vs. simple virilizing) was not available for 3 women and 3 men with CAH.

**Highest level obtained, coded as GCSEs (General Certificates of Secondary Education) = 2; A Levels = 3; Vocational Training = 4; Bachelor = 5; Master = 6.

### Brain image acquisition

Structural T1-weighted brain images were acquired on the same Siemens 3.0 Tesla Skyra system with a 32-channel head coil using the following parameters: TR = 2300 ms, TE = 2.98 ms, flip angle = 9°, matrix = 256 × 240, voxel size = 1 × 1 × 1 mm^3^. The images underwent quality control using visual inspections as well as objective criteria implemented in the CAT12 toolbox^45^. Of the originally acquired 110 brain images, two images from the CAH group did not pass the quality control and were removed. In order to retain the well-matched sample, the corresponding two images of the control group were removed as well, which resulted in a final set of 106 brain images.

### Brain image analyses

Images were processed using MATLAB (https://www.mathworks.com/products/matlab.html) and the CAT12 toolbox^45^, applying bias-field corrections and rigid-body transformations. Using the processed images, the corpus callosum was manually outlined by one rater (C.D.) in each brain’s midsagittal section^46^. Before tracing the current dataset, intra- and inter-rater reliability were established by tracing an independent set of twenty brain images twice by two raters (C.D. and F.K.). Both intra- and inter-rater reliability were high, with dice indices of 0.97 and 0.98 (intra-rater) as well as 0.94 (inter-rater).

**Fig. 1:**
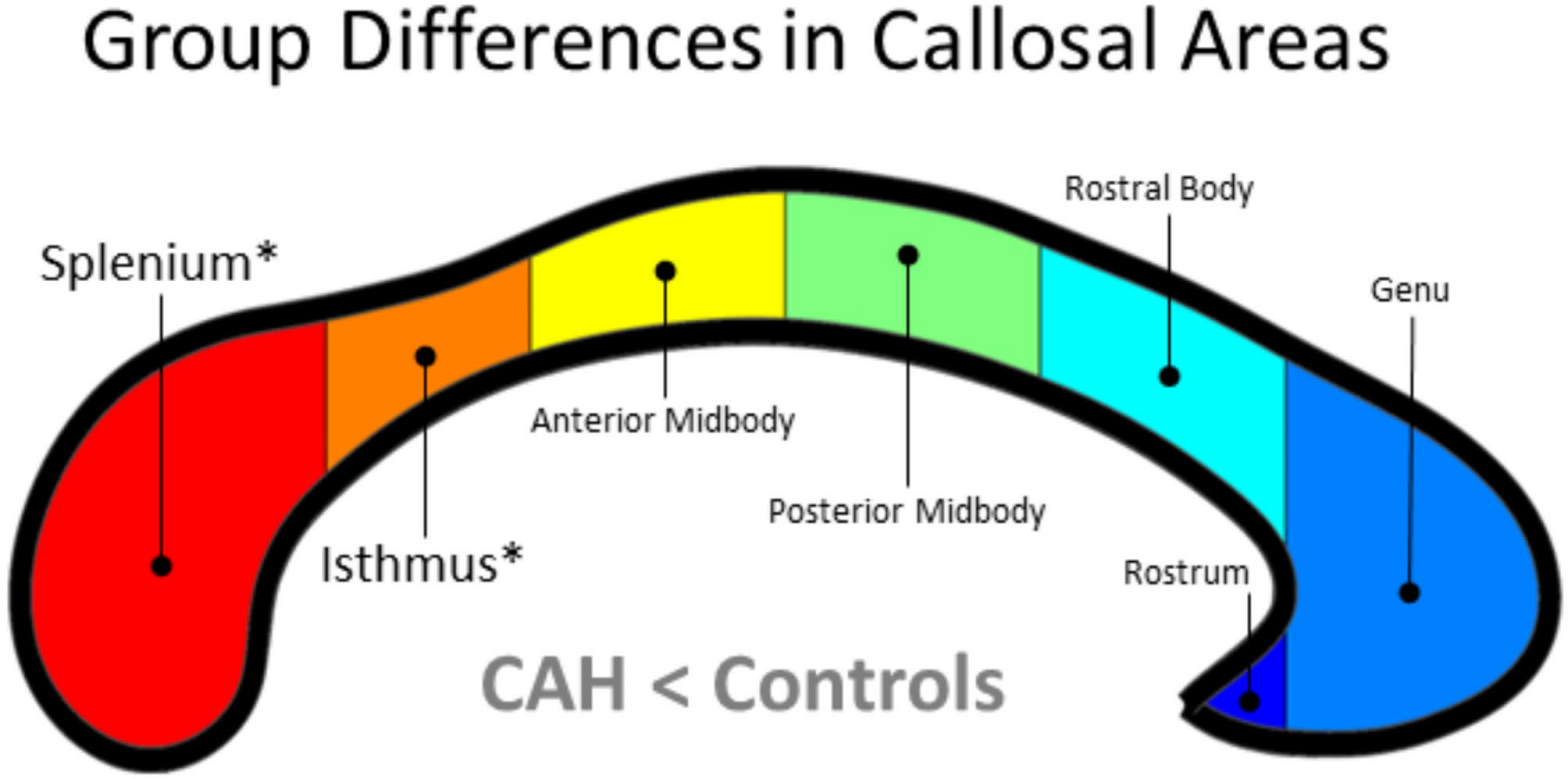
Group differences in callosal areas. Significantly smaller callosal areas in individuals with CAH compared to controls (CAH < Controls) for splenium and isthmus, as indicated with an asterisk. The corpus callosum was parcellated into seven sections according to the Witelson scheme^24^: the splenium (in red), representing the posterior fifth of callosal area; the isthmus (in orange) representing two fifteenths; the posterior midbody (in yellow) and anterior midbody (in green), both representing one sixth of callosal area; and the anterior third, which consisted of the rostral body (in cyan), the genu (in light blue), and the rostrum (in dark blue). The posterior callosal section points to the left; the anterior section points to the right.

To obtain the area measures, the callosal traces were used to define seven callosal sections according to the Witelson parcellation scheme (see Fig. 1)^24^. This was followed by calculating the areas of the splenium, isthmus, posterior midbody, anterior midbody, rostral body, genu, and rostrum. To obtain the thickness estimates, the callosal traces were automatically processed in a number of successive steps^46^. More specifically, the upper and lower callosal traces were separated into 100 nodes and re-sampled at regular intervals. Then, a new midline segment was created by calculating the 2D average from the 100 equidistant nodes representing the upper and the lower callosal boundaries. Finally, the distances between the 100 nodes of the upper as well as the lower callosal boundaries to the 100 nodes of the midline segment were calculated.

In addition to obtaining the callosal measures, the total intracranial volume (TIV) was estimated. This was achieved by classifying images as gray matter (GM), white matter (WM), and cerebrospinal fluid (CSF) and adding the sub-volumes of these compartments (TIV = GM + WM + CSF). TIV was included as a nuisance variable into the statistical model (see next section).

### Statistical analyses

All statistical comparisons were performed using general linear models and conducting analyses of covariance (ANCOVAs). The seven callosal area measures and the 100 callosal thickness estimates were the dependent variables; group (CAH / control), biological sex (female / male), and the group-by-sex interaction were the independent variables; and TIV and age were variables of no interest. Results were corrected for multiple comparisons using Bonferroni corrections for the area measures, and permutation-based family-wise error (FWE) corrections^43,44^ for the thickness measures.

## Results

### Callosal areas

Neither the main effect of biological sex nor the group-by-sex interaction was significant. In contrast, there was a significant main effect of group: Individuals with CAH had a significantly smaller callosal isthmus (F_[1, 100]_ = 9.69; *p* = 0.0024) and splenium (F_[1, 100]_ = 8.32; *p* = 0.0048) than controls (see Fig. 1). Both effects survived Bonferroni corrections (i.e., *p* < 0.007). No callosal area was significantly larger in individuals with CAH than in controls. For area-specific descriptive statistics, both unadjusted and adjusted for age and TIV, see Tables 2 and 3.

**Table 2:** Raw callosal area measures (mean ± standard deviation) in square millimeters (mm^2^).

	Control women	Control men	Women with CAH	Men with CAH
Rostrum	18.9 ± 8.0	22.8 ± 7.0	17.8 ± 9.6	20.0 ± 8.4
Genu	142.9 ± 28.0	165.5 ± 29.9	141.7 ± 28.3	151.1 ± 27.7
Rostral body	89.4 ± 15.4	102.3 ± 15.3	87.9 ± 13.3	99.1 ± 13.6
Anterior midbody	82.4 ± 10.9	90.8 ± 14.4	80.6 ± 13.2	85.6 ± 11.6
Posterior midbody	76.4 ± 11.0	86.4 ± 12.5	72.6 ± 12.1	79.0 ± 10.4
Isthmus	63.0 ± 10.9	74.9 ± 18.3	55.0 ± 11.8	63.3 ± 15.4
Splenium	196.9 ± 25.5	218.0 ± 30.1	177.0 ± 29.7	196.4 ± 38.0

**Table 3:** Residual callosal area measures (mean ± standard deviation) in square millimeters (mm^2^) (adjusted for age and TIV.).

	Control Women	Control Men	Women with CAH	Men with CAH
Rostrum	19.2 ± 7.7	21.6 ± 7.2	18.7 ± 9.1	19.2 ± 8.5
Genu	148.4 ± 26.5	150.9 ± 26.9	151.2 ± 25.0	141.0 ± 21.3
Rostral body	91.5 ± 15.4	96.7 ± 12.9	91.5 ± 12.6	95.2 ± 12.3
Anterior midbody	84.5 ± 10.5	85.0 ± 13.2	84.4 ± 11.9	81.6 ± 9.3
Posterior midbody	78.3 ± 10.5	81.5 ± 12.0	75.7 ± 11.4	75.5 ± 8.0
Isthmus	64.7 ± 10.9	70.1 ± 17.4	58.1 ± 11.0	60.1 ± 13.9
Splenium	202.3 ± 23.7	203.0 ± 25.5	186.9 ± 27.8	186.1 ± 31.7

### Callosal thickness

**Fig. 2:**
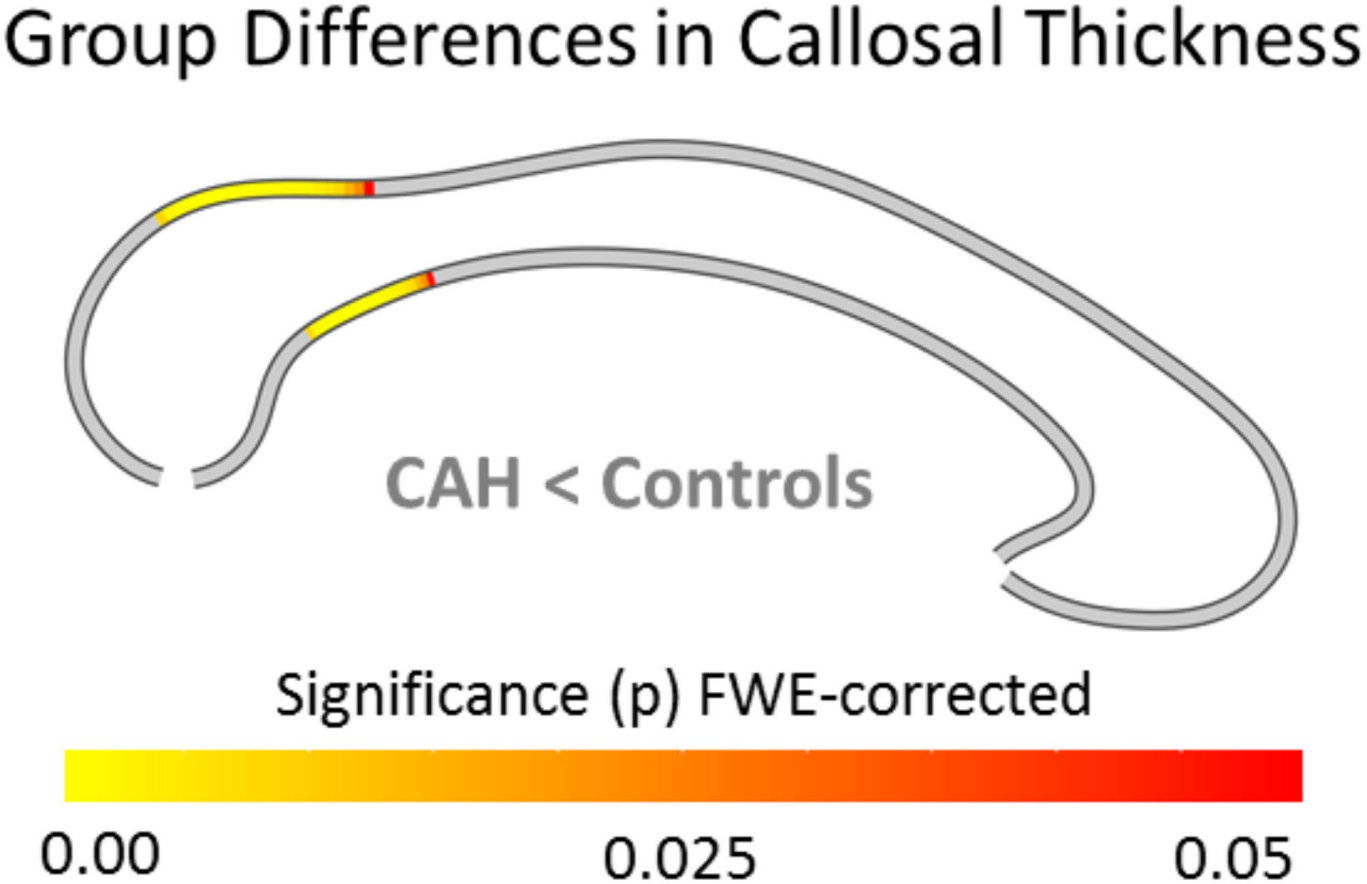
Group differences in callosal thickness. Significantly thinner corpora callosa in individuals with CAH compared to controls (CAH < Controls) within splenium and isthmus. The color bar encodes significance (p) at FWE-corrected levels. The posterior callosal section points to the left; the anterior section points to the right.

Neither the main effect of biological sex nor the group-by-sex interaction was significant. However, as shown in Fig. 2, individuals with CAH had a significantly thinner corpus callosum within splenium and isthmus than controls mirroring the significant effects for the area measures. No callosal region was significantly thicker in individuals with CAH than in controls.

## Discussion

To our knowledge, this is the first study explicitly focusing on the corpus callosum in a large sample of individuals with CAH and well-matched controls. Both the callosal area and thickness analyses revealed a significantly reduced splenium and isthmus in CAH. In terms of the direction of the effect, our findings corroborate previous studies highlighting white matter deficits in general^8–19,47^, and callosal alterations in particular^10,13–15,17,20–22^, in CAH. Given that the corpus callosum connects the two hemispheres, our findings might also relate to prior reports of gray matter deficits in CAH, particularly in posterior brain regions (e.g., within the parieto-occipital lobe), as reviewed elsewhere^19^.

The underlying cause for the observed variations in the corpus callosum remains to be determined. Given that we excluded participants with neurological or psychiatric disorders, the observed callosal reductions are not a consequence of any co-morbidity. Moreover, given that CAH causes elevated androgen levels in female fetuses but not male fetuses, the lack of a significant group-by-sex interaction indicates that callosal reductions are not due to prenatal androgen exposure^8,48,49^. Instead, given the significant main effect of CAH, the observed callosal reductions could be a consequence of the condition itself, encompassing not only direct biological or neurological impacts but also indirect influences through altered social environments and experiences (e.g., minority stress). Furthermore, given that both women and men with CAH receive exogenous glucocorticoids, it could be that the treatment has caused (or at least contributed) to the observed effects in the corpus callosum. In agreement with this assumption, prior research^50^ revealed that clinically appropriate treatment of pregnant sheep with repeated courses of corticosteroids significantly delayed myelination of the corpus callosum in newborns (i.e., there were fewer myelinated axons, more unmyelinated axons, reduced axon diameters, and thinner myelin sheaths). Granted, outcomes from animal studies are not directly transferrable to humans, and corticosteroids in that study were administered to the mother and before birth, rather than to the offspring and after birth (i.e., as usually done in individuals affected by CAH). Nevertheless, elevated or abnormal glucocorticoid levels have also been reported to amplify neurotoxic insults or disrupt neuronal myelination in humans^9^. Thus, it is possible that the observed callosal reductions are a consequence of glucocorticoid therapy.

In terms of the location of the effects, our findings are in agreement with prior reports of focal lesions and white matter hyperintensities in CAH within the callosal splenium^13,20,21^. Pronounced alterations in this most posterior callosal segment could be explained by callosal ontogeny: As reviewed elsewhere^51^, there have been two main theories regarding callosal development in utero: The prevalent theory is that callosal axons first cross the midline toward the anterior end and then towards the posterior end, albeit recent neuroimaging studies of human embryology seem to suggest that initial callosal connections emerge more centrally and then subsequently progresses bi-directionally both anteriorly and posteriorly, but with more prominent growth anteriorly. Either way, the callosal splenium (situated most posteriorly) as well as the isthmus (located adjacent to the splenium) might be among the last callosal regions to develop. Thus, given that corticosteroid treatment of infants with CAH often begins shortly after birth, those regions might be the most vulnerable to effects of corticosteroids.

Follow-up studies might add to the current findings by relating individual cortisol levels and perhaps also androgen levels (neither information was available for the current cohort) to the callosal measures. Moreover, expanding the size of the salt-wasting cohort (the current study only contained 20 women and 9 men with this phenotype) and conducting cohort-specific analyses might reveal additional clues on whether early salt-wasting crises might have contributed to the observed callosal variations in individuals with CAH. Last but not least, future studies might benefit from extending the imaging battery from T1-weighted images to T2-weighted, diffusion-weighted, and/or FLAIR images as those are particularly sensitive to any white matter alterations.

## Data Availability

The data are not publicly available due to ethical restrictions imposed by the signed consent. Any reasonable request for data access should be made to the corresponding author.
